# Comparison of analyses of the QTLMAS XII common dataset. I: Genomic selection

**DOI:** 10.1186/1753-6561-3-s1-s1

**Published:** 2009-02-23

**Authors:** Mogens Sandø Lund, Goutam Sahana, Dirk-Jan de Koning, Guosheng Su, Örjan Carlborg

**Affiliations:** 1 Aarhus University, Faculty of Agricultural Sciences, Department of Genetics & Biotechnology, Research Centre Foulum, DK-8830, Box 50, Tjele, Denmark; 2 The Roslin Institute and R(D)SVS, University of Edinburgh, Roslin Biocentre, Roslin, Midlothian, EH25 9PS, UK; 3 Department of Animal Breeding and Genetics, Swedish University of Agricultural Sciences, Box 7023, SE-75007 Uppsala, Sweden

## Abstract

A dataset was simulated and distributed to participants of the QTLMAS XII workshop who were invited to develop genomic selection models. Each contributing group was asked to describe the model development and validation as well as to submit genomic predictions for three generations of individuals, for which they only knew the genotypes. The organisers used these genomic predictions to perform the final validation by comparison to the true breeding values, which were known only to the organisers. Methods used by the 5 groups fell in 3 classes 1) fixed effects models 2) BLUP models, and 3) Bayesian MCMC based models. The Bayesian analyses gave the highest accuracies, followed by the BLUP models, while the fixed effects models generally had low accuracies and large error variance. The best BLUP models as well as the best Bayesian models gave unbiased predictions. The BLUP models are clearly sensitive to the assumed SNP variance, because they do not estimate SNP variance, but take the specified variance as the true variance. The current comparison suggests that Bayesian analyses on haplotypes or SNPs are the most promising approach for Genomic selection although the BLUP models may provide a computationally attractive alternative with little loss of efficiency. On the other hand fixed effect type models are unlikely to provide any gain over traditional pedigree indexes for selection.

## Introduction

### Genomic selection

Hybrid Marker Assisted Selection (MAS) schemes were the first tool proposed to include information on a few main genes or quantitative trait loci (**QTL**) into best linear unbiased prediction (**BLUP**) of breeding values e.g. [1]. More recently, genomic selection (**GS**) [2] was proposed. This approach relies on a genome-wide dense marker map, such that markers in linkage disequilibrium (**LD**) with each QTL are available. GS hence utilizes the data on all available markers to produce genomic estimated breeding values (**GEBV**) by summing the effects of all small chromosome segments characterized by their marker alleles. Because predictions using GS are based on marker associations and not pedigree information, the requirement to have phenotypes on selection candidates or their close relatives is relaxed and a breeding value can be obtained as soon as the genotypes are available. As a result, the method has the potential to increase genetic progress as well as reducing costs [3]. In breeding schemes with long generation intervals (e.g. cattle) the increase will mainly be a consequence of shortening the interval. In breeding schemes with shorter generation intervals the genetic gain may be increased due to higher accuracies of breeding values at the time of selection. With new genotyping technologies becoming available for livestock species (eg. Bovine50 Bead Chip ), GS is now becoming a very attractive approach to predict breeding values.

Several statistical methods have been proposed to be used in genomic prediction models [2,4]. Apart from the specific models used, the prediction of genomic breeding values also involve steps of data editing, choice of response variable, utilization of marker information, and model validation.

The aim of this study was to compare approaches used to predict genomic breeding values in a situation that mimics real life. This was done by distributing genotypic data, phenotypic data, and pedigree information, but not the true breeding values to participants of the QTL-MAS XII workshop. Participants then applied various approaches to predict genomic breeding values for three generations of non-phenotyped individuals. The organisers then compared the predictions with true simulated breeding values.

## Methods

### Simulation of data

#### Pedigree

**Figure 1:**
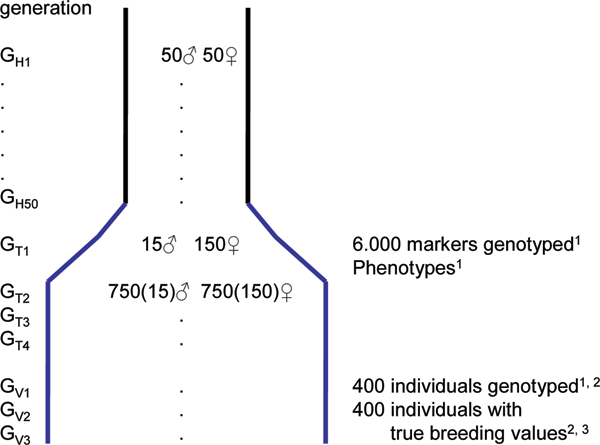
**Design of the simulation study**. ^1^Data provided to participants. ^2^400 individuals sampled randomly in each generation from population of 1500. ^3^True breeding values known only to organisers for validation. Numbers in parenthesis is the number of parents for the next generation.

The pedigree was simulated in three parts as illustrated in Figure 1. First a historic population was simulated with 50 generations without records (G_h1 _to G_h50_), followed by 4 generations (G_t1 _to G_t4_; the training set), with both genotype and phenotype records, and finally the last 3 generations (G_v1 _to G_v3_; the validation set) were only genotypes and true breeding values were generated. The historic population was created by 100 founder individuals (50 males and 50 females) in generations G_h1_. For each of the subsequent 50 generations, 50 males and 50 females were produced by randomly sampling parents from the previous generation. The base generation of the recorded pedigree (G_t1_) had 15 males and 150 females. The parents for these were sampled randomly from individuals in G_h50_. Each male was mated to 10 females and each mating pair produced 10 offspring. This created a fullsib-halfsib design, in which each male had 100 progeny and each female had 10 progeny and a total of 1500 individuals per generation. In the following 5 generations (G_t2 _to G_t4 _and G_v1 _to G_v2_), 15 males and 150 females were selected randomly to be parents of the next generation and the same mating design was repeated. In the last 3 generations (G_v1 _to G_v3_) 400 of the 1500 individuals were selected randomly to be genotyped. The resulting 1200 individuals constitute the validation set.

#### Marker and QTL

Marker alleles were sampled for 6000 biallelic loci on 6 chromosomes (1000 markers on each chromosome) with 0.1 cM between adjacent loci. In the founder individuals (G_h1_), the two alleles at each marker locus were sampled with equal probabilities. Recombination was sampled according to Haldane's mapping function [5]. A total of 48 QTL were simulated. QTL positions were sampled under the assumption of a multinomial distribution of genes across the genome. The multinomial distribution used was based on the genetic map of the mouse genome [6]. The allele substitution effects of the QTL were drawn from a gamma distribution with scale parameter (α) 5.4 and shape parameter (β) 0.42 following [7]. We wanted to control the genetic effects and genomic location of four QTL. To achieve this, we replaced four of the 48 QTL that were closest to the desired locations with QTL of pre-defined effects. The allele substitution effects of these four fixed QTL were standardized based on their individual allelic frequencies in the last generation of the historic pedigree, so that each of these QTL explains a predefined percentage of the genetic variance. Positions and allele substitution effects and contribution to genetic and phenotypic variance for each QTL is available at 

#### Phenotype

The phenotypes were obtained as the cumulative effects of the 44 randomly drawn QTL, the 4 defined QTL, and a random residual. First, the effects of the 44 random-QTL were summed. The effect was standardized to mean 0 and variance 1. Then the effects of four fixed QTL were added to constitute the total genetic effect. The residual variance was defined to obtain a heritability of 0.30. The individuals' phenotypes were derived as the sum of the individuals' genetic value and a random residual drawn from a normal distribution with mean zero and variance equal to the residual variance.

### Validation of prediction models

Participants of the QTLMAS XII workshop were provided pedigree, phenotypic, and genomic data on the 4665 individuals of generations G_t1 _to G_t4 _and only pedigree and genomic data on generations G_v1 _to G_v3_. Using these data, they used various models to estimate GEBVs for individuals in generations G_v1 _to G_v3_. The properties of the reported GEBVs were then assessed by three different criteria to relate the GEBVs reported by the workshop participants and the true breeding values (TBV), which were only known by the organisers of the workshop.

The first criterion was the accuracy of the GEBVs as a measure of their predictive ability. Accuracies were calculated as the correlation between GEBVs and TBVs. The second criterion was the bias of the GEBVs, assessed as the coefficients of regressing TBVs on GEBVs. A regression coefficient close to 1 indicates that predictions are unbiased. The third criterion was the rank correlations between GEBVs and TBVs in the top 10% of individuals ranked on TBVs.

### Genomic selection models

A range of different statistical methods were used by QTLMAS XII participants to analyse the simulated data. Some participants have provided GEBVs from all their investigated models to the organisers while some have provided GEBVs from selected models only. According to the properties of the models for which GEBVs have been provided, they can be classified into three categories: 1) fixed effect models, 2) BLUP based models with random marker effects, and 3) Bayesian MCMC based models with a mixture of some markers attributed a large variance and the majority attributed a small variance. Different characteristics of the models used and relation to the model specifications in the contributed papers are shown in Table 1 for the fixed effect models, in Table 2 for the BLUP based models, and in Table 3 for the Bayesian models. Except for models GP_BLUP11_, GP_BLUP12_, and GP_BLUP16 _the provided phenotypes were used as the response variable in the analyses. Only models GP_Bayes1 _and GP_Bayes3 _included a polygenic effect in the model and only models GP_Bayes1_, GP_Bayes3_, and GP_Bayes5 _used haplotype information.

**Table 1:** Fixed effects models, their name in the contributed paper, and number of SNPs fitted.

**Model**	**Model [reference]**	**Number of markers**
GP_Fix1_	GBV(1)6000 [11]	6000
GP_Fix2_	GBV(1)3328 [11]	3328
GP_Fix3_	GBV(1)1200 [11]	1200
GP_Fix4_	GBV(1)600 [11]	600
GP_Fix5_	GBV(1)300 [11]	300

**Table 2:** Random effects BLUP models, their name in the contributed paper, assumed SNP variance and response variable used.

**Model**	**Model [reference]**	**Number of markers**	**SNP variance**	**Respons variable**
GP_BLUP1_	GBV(3)3328 [11]	3328	σ^2^_G_	Phenotype
GP_BLUP2_	GBV(3)1200 [11]	1200	σ^2^_G_	Phenotype
GP_BLUP3_	GBV(3)600 [11]	600	σ^2^_G_	Phenotype
GP_BLUP4_	GBV(3)300 [11]	300	σ^2^_G_	Phenotype
GP_BLUP5_	GBV(4)3328 [11]	3328	σ^2^_G_/3328	Phenotype
GP_BLUP6_	GBV(4)1200 [11]	1200	σ^2^_G_/1200	Phenotype
GP_BLUP7_	GBV(4)600 [11]	600	σ^2^_G_/600	Phenotype
GP_BLUP8_	GBV(4)300 [11]	300	σ^2^_G_/300	Phenotype
GP_BLUP9_	GEBV1 [14]	595	σ^2^_G_	Phenotype
GP_BLUP10_	GEBV2 [14]	595	σ^2^_G_/595	Phenotype
GP_BLUP11_	GEBV3 [14]	618	σ^2^_G_	EBV
GP_BLUP12_	GEBV4 [14]	618	σ^2^_G_/618	EBV
GP_BLUP13_	BLUP1 [15]	6000	σ^2^_E_	Phenotype
GP_BLUP14_	BLUP2 [15]	6000	σ^2^_G_/6000	Phenotype
GP_BLUP15_	RR2* [15]	6000	Ridge coefficient	Phenotype
GP_BLUP16_	RR2* [15]	6000	Ridge coefficient	EBV

**Table 3:** Bayesian models, their name in the contributed paper, assumptions on SNP effects and polygenic effects.

**Model**	**Model [reference]**	**SNP effect**	**Polygenic effect**	**Number of QTL assumed apriori**
GP_Bayes1_	HAP_POL [16]	IBD	+	30
GP_Bayes2_	HAP_NOPOL [16]	IBD	-	30
GP_Bayes3_	SNP_POL [16]	Single SNP	+	30
GP_Bayes4_	SNP_NOPOL [16]	Single SNP	-	30
GP_Bayes5_	Scenario11 [17]	5 SNP haplotype	-	12

## Results and discussion

**Figure 2:**
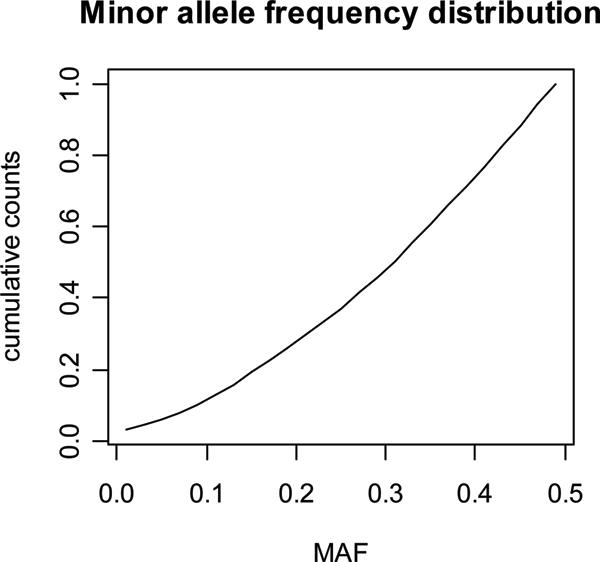
**Cumulative distribution of minor allele frequencies in the last 7 generations**.

In generations 1–7, the mean minor allele frequency (**MAF**) of markers was 0.298. The cumulative distribution of MAF in Figure 2 shows a rather equal distribution, which is a consequence of random drift over the 50 generations from the original starting values of 0.5 for each locus. For 38 marker loci one allele was fixed. The mean linkage disequilibrium (r^2^) between adjacent SNPs was 0.20 and the median was 0.11. This may be relatively low compared to other simulation studies that often use 1000 generations of random mating. On the other hand, the LD achieved in this paper seems very comparable to the realised values from real data analysis. For markers with a distance around 0.1 Mb the average R2 was 0.14 and 0.22 in different breeds and studies [8-10].

### Fixed effect models

Only one contribution [11] used fixed effects models to predict GEBVs of the simulated data. They used 4 different sets of SNPs in the prediction models ranging from using all 6000 SNPs, every 5^th ^SNP, every 10^th ^SNP, every 20^th ^SNP or setting a MAF of 0.2. As expected, the model fit improved as more SNPs were fitted in the training set [11]. However, the predictive ability of the models in the validation dataset decreased with more SNPs in the model (Table 4). Using all 6000 SNPs resulted in a correlation between GEBVs and TBVs (averaged over generations G_v1 _to G_v3_) of 0.16 compared to a correlation of 0.56 with 300 SNPs.

**Table 4:** Comparison of genomic estimated breeding values (GEBV) and true breeding values (TBV) for fixed effects models.

**Model**	**Accuracy^1^**	**Regression coefficient^2^**	**Rank correlation^3^**
GP_Fix1_	0.16	0.03	-0.10
GP_Fix2_	0.23	0.06	0.13
GP_Fix3_	0.48	0.28	0.29
GP_Fix4_	0.54	0.41	0.32
GP_Fix5_	0.56	0.54	0.37

^1^Accuracy of GEBV measured as correlation between GEBV and TBV. ^2^Regression of GEBV on TBV as a measure of bias. ^3^Rank correlation between TBV and GEBV for individuals in the top 10% TBV rank.

All evaluated fixed effects models overestimated breeding values severely (Table 4). The regression coefficients of TBVs on GEBVs were between 0.03 and 0.53 averaged over generations G_v1 _to G_v3_. The biases increased with the number of markers in the model and were particularly strong when all SNPs were used. Table 4 shows that the rank correlations drop dramatically with the inclusion of more markers in the model. This means that the low regression coefficient for this model was not only due to a scaling effect. In the worst case more parameters are fitted than available observations, which lead to a serious over-fitting of data. This is known to result in unstable prediction with large prediction errors [12]. As a result, the over-fitted model had poor predictive ability for the data beyond the training set, resulting in a large variance of GEBVs. These results are well in line with the original observation of [2], where it was found that using a least squares approach leads to biased GEBVs and poor predictive ability. On the other hand [13] found relatively good results using fixed regression models, if markers were selected based on associations to the phenotypes and a liberal significance threshold. This indicates that fixed effects models could be more useful that the current simulations indicated, if a procedure is used that balance the problems of over-fitting data and selecting SNPs with overestimated effects.

### Random effect models

Three contributions presented GEBVs from BLUP models fitting SNPs as random effects. In two studies [11,14] the variance explained by each SNP was given as either the total genetic variance (σ^2^_G_) or the total genetic variance divided by the number of SNPs (σ^2^_G_/N_SNPs_). It was apparent that models using σ^2^_G _lead to results similar to those from the fixed effects models. This is most apparent when comparing least squares models GP_Fix2 _– GP_Fix5 _([11]; Table 4) to BLUP models GP_BLUP1 _– GP_BLUP4 _([11]; Table 5), where correlations and regression coefficients are very similar when the same number of SNPs were used. When σ^2^_G_/N_SNPs _was used instead, the models performed much better. This is apparent when comparing accuracies from models GP_BLUP1 _to GP_BLUP4 _with those from models GP_BLUP5 _to GP_BLUP8_. The correlation between GEBVs and TBVs increased from 0.23 to 0.75 when 3328 SNPs were used (GP_BLUP1 _and GP_BLUP5_) and from 0.56 to 0.61 when only 300 SNPs were used (GP_BLUP4 _and GP_BLUP8_). When using this variance, the SNP effects are regressed considerably more towards zero, which leads to virtually unbiased estimates for the models with more than 600 SNPs.

**Table 5:** Comparison of genomic estimated breeding values (GEBV) and true breeding values (TBV) for BLUP models.

**Model**	**Accuracy**	**Regression coefficient**	**Rank correlation**
GP_BLUP1_	0.23	0.06	0.14
GP_BLUP2_	0.49	0.29	0.28
GP_BLUP3_	0.52	0.39	0.31
GP_BLUP4_	0.58	0.56	0.38
GP_BLUP5_	0.75	0.99	0.40
GP_BLUP6_	0.73	1.07	0.45
GP_BLUP7_	0.71	1.01	0.46
GP_BLUP8_	0.61	0.88	0.44
GP_BLUP9_	0.55	0.41	0.20
GP_BLUP10_	0.77	0.94	0.35
GP_BLUP11_	0.55	1.14	0.19
GP_BLUP12_	0.53	1.36	0.25
GP_BLUP13_	0.22	0.06	-0.02
GP_BLUP14_	0.51	0.31	0.22
GP_BLUP15_	0.49	0.29	0.21
GP_BLUP16_	0.45	0.85	0.17

^1^Accuracy of GEBV measured as correlation between GEBV and TBV. ^2^Regression of GEBV on TBV as a measure of bias. ^3^Average squared difference between GEBV and TBV. ^4^Rank correlation between TBV and GEBV for individuals in the top 10% TBV rank.

The effect of the assumed variance explained by each SNP on the accuracy of GEBV can also be seen in the other two contributions. In the contribution [14] GEBV were obtained from a BLUP model with the variance either σ^2^_G _or σ^2^_G_/N_SNPs_. Here the correlation between TBVs and GEBVs increased from 0.55 to 0.77 and the regression coefficient increased from 0.41 to 0.94, when phenotypic values were used as the response variable. In the study by Pimentel *et al*. [15], the correlation was 0.22 and the regression was 0.06 using a model with the variance of each SNP equal to residual variance, while the correlation was 0.51 and the regression was 0.31 using a model with the variance σ^2^_G_/N_SNPs_. These results clearly show that the importance of fitting the SNP effects as random effects and providing a reasonable SNP variance increases with the number of markers included in the models.

### Mixture distribution/Bayesian models

Two contributions [16,17] analysed data with Bayesian methods and in total submitted GEBVs from five different models. GEBVs from all models were more accurate (Table 6) than the non-Bayesian models. All models had a high predictive ability with correlations between GEBVs and TBVs ranging from 0.84 to 0.92 averaged over generations G_v1 _to G_v3_.

**Table 6:** Comparison of genomic estimated breeding values (GEBV) and true breeding values (TBV) for Bayesian models.

**Model**	**Accuracy**	**Regression coefficient**	**Rank correlation**
GP_Bayes1_	0.84	0.85	0.46
GP_Bayes2_	0.84	0.86	0.48
GP_Bayes3_	0.86	0.94	0.56
GP_Bayes4_	0.87	0.96	0.56
GP_Bayes5_	0.92	0.98	0.53

^1^Accuracy of GEBV measured as correlation between GEBV and TBV. ^2^Regression of GEBV on TBV as a measure of bias. ^3^Average squared difference between GEBV and TBV. ^4^Rank correlation between TBV and GEBV for individuals in the top 10% TBV rank.

All Bayesian models used Gibbs sampling algorithms, in which marker or QTL effects were assumed to follow a mixture distribution, where relatively few markers were assumed to explain a large variance and a large number explained a very small variance. This is most likely the main reason for the improved performance of these models over the BLUP models in which all markers are assumed to explain the same amount of variance. The assumption of a homogeneous variance for all markers leads to a poor prediction of the effect of a QTL with a large contribution to the trait from a single marker even if they are in complete LD. In this simulated dataset the 10 largest QTL explain 82.9% of the genetic variance. This may favour the Bayesian models relative to BLUP models, compared to situations with a large number of QTL contributing more equally to the genetic variance.

### Fitting single SNP or haplotype effects

Following the internal validation by [17], it can be seen that, for this data, it was an advantage to fit effects of haplotypes rather than effects of single SNPs with the model used in [17]. It must be noted that the data were provided with known haplotypes. In real life, haplotypes are estimated with errors, which may affect the results. The advantage of using haplotypes in this study is most likely because there is higher LD between the haplotypes and the QTL than between any of the individual markers and the QTL. On the other hand there are some disadvantages of fitting haplotype effects. These disadvantages include: 1) for a given position there are more effects to be estimated, 2) large haplotypes are more likely to break up by recombinations, 3) haplotypes are more sensitive to errors in the map. Therefore, the optimal size of haplotypes is a trade-off between having a predictor in high LD with the underlying genes and the precision to estimate haplotype effects [18].

Models GP_Bayes1 _and GP_Bayes2 _fit haplotype effects with a correlation between alleles proportional to the probability of IBD. This is expected to perform better as more phenotypes contribute to the estimation of particular haplotype effects. However, Table 6 shows that the accuracies are slightly worse and regressions further from 1 compared with the other Bayesian models. In particular, it seems that the predictive ability, both assessed as accuracies and the rank correlations for the top animals, decreases faster over generations. A possible explanation is that the IBD based model is theoretically advantageous, but it may be associated with numerical problems. For instance, the IBD matrices calculated from pair wise comparisons of haplotypes are generally not positive definite and must be manipulated before inversion.

### Inclusion of polygenic effects

Comparing GP_Bayes1 _to GP_Bayes2 _and GP_Bayes3 _to GP_Bayes4 _(Table 6) shows no effect of including a polygenic component in the model for these analyses. However, we do not know what proportion of genetic variance can be captured by SNP markers in real data. If SNP markers don't capture all the genetic variance it is still important to include a polygenic component. In the present study, the total genetic effect was simulated from bi-allelic QTL and the markers were uniformly distributed across the whole genome. However, in real data, part of the genetic variance comes from structural variation such as copy number variation, inversion, deletion etc. [19]. Also the simulated population is very homogeneous. In real data there are likely to be genetic structures that lead to spurious associations [20]. In such situations inclusion of pedigree may improve predictions more that what is observed in this simulated dataset.

### Using EBVs or phenotypic values as response variables

Two of the contributions used both phenotypic values and EBVs as response variables in the analyses. In the study by Pimentel *et al. *[15], the correlation between TBV and GEBV derived from phenotypic values was slightly higher than the correlation for GEBV derived from EBVs. In the study by Macciotta *et al. *[14], using a model with SNP variance of σ^2^_G_/N_SNPs_, the accuracy of GEBV based on EBVs was 0.53, but the accuracy was 0.77 for GEBV based on phenotypic values. The lower accuracy of GEBV obtained using EBVs as response variable is most likely due to information lost in the procedure of predicting breeding values, which do not have high accuracies themselves. If this is the case, EBVs should only be used as response variables when they have very high reliabilities. The same effect is seen in the models by [15] who also used both EBVs and phenotypes as the response variable.

### Model validation

Several workshop participants performed internal validations by either estimating correlations between GEBVs and phenotypes or EBVs in the training set or by cross validation. When correlations are calculated within the training set only, this reflects mainly how well the model fit the data in the training set and not necessarily how well the model predict the next generation. When a statistical model includes too many covariates, relative to the amount of data available, the model may fit the data perfectly but have a poor predictive ability. This was most obvious in the results obtained using fixed effects models. In [11], the correlation between EBV and GEBV in the training set was highest when all markers were included and declined as markers were removed. The predictive ability was, however, actually very poor for the fixed model with all markers (GP_Fix1_) and increased as markers were removed from the model (GP_Fix2 _– Gp_Fix5_).

To assess the predictive ability, it is necessary to perform model validation. There are many approaches to model validation. A common approach in statistical practice is cross-validation where data sample is partitioned into subsets. The analysis is then initially performed on a single of these subsets, while the others are retained for subsequent validation of the initial analysis. In the workshop, [15] validated the models using data of G_t1_-G_t3 _as training data and G_t4 _as test data. This approach may have the disadvantage of using EBVs for validation of GEBVs in G_t4 _that were based on information from phenotypes of individuals in G_t1_-G_t3_. This creates a strong dependency between the data used for model development and validation, which could be avoided by using the phenotypic data in generation G_t4_ rather than EBVs. Villumsen *et al. *[17] used a 5-fold cross validation where in each of the five validation sets, 20% of the data in G_t4_, were taken as test data. The advantage of cross validation is that it makes it possible to retain training data as large as possible, while obtaining the amount of total test data as large as required (with maximal total test data equal to the whole data). In the simulated data of this workshop this strategy proved very efficient in selecting models that predicted the TBVs in the validation data very accurately.

### Decrease of accuracies over generations

While three generations were simulated for validation, results are given as the mean accuracy of all three generations. This is because we could see no clear trend as to which differences in the models lead to a higher decrease in accuracy over the three generations. The only obvious result was a clear relation between the accuracy of GEBVs in generation G_v1 _and the decline from G_v1 _to G_v3_, such that models with a high accuracy declined less than models with low accuracies.

It must be noted that the current comparison is based on a single replicate of simulated data and therefore the conclusions must be interpreted with caution.

## Conclusion

The comparison of the different methods applied to the dataset by the workshop participants clearly shows a distinct clustering of the three approaches, where the Bayesian analyses gave the highest accuracies, followed by the BLUP models, while the fixed effects models generally had low accuracies and large error variance. However, some BLUP models were less biased than some Bayesian models. The BLUP models are clearly sensitive to the given SNP variance, because a BLUP does not estimate SNP variance, but takes the specified variance as the true variance. For instance, if the number of QTL would increase, and each QTL would have a smaller effect, it is expected that the differences between the BLUP and Bayesian models would be smaller. The current comparison suggests that Bayesian analyses on haplotypes or SNPs are the most promising approach for Genomic selection although the BLUP models may provide a computationally attractive alternative with little loss of efficiency. As already concluded by [2] fixed effect type models are unlikely to provide any gain over traditional pedigree indexes for selection.

## List of abbreviations used

GEBV: genomic estimated breeding values; GS: genomic selection; TBV: true breeding value.

## Competing interests

The authors declare that they have no competing interests.

## Authors' contributions

MSL, ÖC, and DJK designed the simulations. GSa and MSL wrote simulation software. GSa performed the simulations and computed correlations to true breeding values. MSL drafted the manuscript and all authors contributed to interpretation of results.
